# Professional Identity among New Rehabilitation Graduates in China: A Cross-Sectional Questionnaire-Based Survey

**DOI:** 10.4314/ejhs.v33i5.16

**Published:** 2023-09

**Authors:** Xue Zhang, Kai-Yi Qiu, Shao-Zhen Chen

**Affiliations:** 1 School of Rehabilitation and health care, Guangzhou Health science college, Guangzhou, China; 2 Department of Hand-foot Rehabilitation, Guangdong Work Injury Rehabilitation Hospital, Guangzhou, China; 3 Department of Rehabilitation Medicine, Sun Yat-sen University First Affiliated Hospital, Guangzhou, China

**Keywords:** Education, employment, professional identity, questionnaire, rehabilitation

## Abstract

**Background:**

The rehabilitation industry suffered in a terrible economic climate caused by the coronavirus disease 2019 (COVID-19). Healthcare resources and the labor force were directed towards epidemic prevention of post-pandemic, which exacerbated the issue. This study evaluated the professional identity (PI) of new graduates majoring in rehabilitation therapy during the final harsh phase of COVID-19 and explored the factors influencing PI.

**Methods:**

A cross-sectional investigation with a questionnaire was used in this study. A convenience sample of new graduates who majored in rehabilitation were recruited from hospitals and universities nationwide. The data collected demographic information and scores of professional identities for students in the final phase of the strict COVID-19 control policy in 2022.

**Results:**

This study indicated that the average PI was quite a distance from the degree of ‘agree’ (3.84 on average), particularly for the fitness dimension (3.64 on average). The results identified two influencing variables: employment (Beta= -0.09, P< 0.05) and educational patterns (Beta= 0.12, P< 0.01).

**Conclusions:**

Higher education in rehabilitation should respond to the present shift in the structure of the rehabilitation industry post-COVID-19. Meanwhile, the occupational environment of rehabilitation therapists and the medical value of rehabilitation therapy deserves more attention. This study provides evidence for managers to improve organizational justice and adjust policies on the distribution of medical resources.

## Introduction

The corona virus disease 2019 (COVID-19) has increased global economic uncertainties (1,2). Recognizing them provides an important context in analyzing the impact on the healthcare system, the effectiveness of redistribution of resources and workforce, and the rehabilitation trajectory in the post-pandemic era. It is crucial to comprehend how COVID-19 had exacerbated a preexisting malaise in higher education, how it affected new graduates, and what might be done to solve these issues, while simultaneously managing the rehabilitation industry's recovery.

Professional identity (PI) has been defined precisely and clearly by extensive research (3). It is a complex, integrative process of knowledge, beliefs, attitudes, personal norms and values that reflects the needs of the profession and the workplace, in addition to the fundamental values and standards of the profession (4). It addresses the questions of who I am, how well I understand my major, and whether I demonstrate emotional and behavioral dedication to my major (5). Professional identity is dynamic and can be impacted by a wide range of factors such as professional circumstances, sociocultural background, and economic situation (6). The performance of PI may be affected, and might require further attention when confronted with a significant social event or transition, including a pandemic.

This interest in professional identity is partially a product of government programs designed to evaluate how well higher education institutions are capable of meeting broader economic demands (7). Basically, PI is significant for higher education students to find the motivation to learn, put effort in performance and be ready to face adversity, and find the willingness to engage in profession-related work after graduation. Moreover, it impacts on students' career performance later in life, which relates to individual perceptions and evaluations of their progress in the context of their ambitions (8). In sum, PI influences the future plans and career decisions of students in addition to their university academic performance. Consequently, PI serves as an indicator that determines the direction of educational reform and programs that prepare students for the workforce (9,10).

The assessment of professional identity among students majoring in a variety of professions, including medicine and nursing (9,11,12), occupational therapy (13), pharmacy (14), had garnered considerable attention in a number of countries. An earlier study (15) on the PI of Chinese medical students after COVID-19 showed that there were significant differences among majors, which were grouped into three categories: clinical, nursing, others. The ‘clinical’ scores (3.44 ± 0.37) ranked first, while the ‘other’ (3.33 ± 0.44) stood at the lowest scores. Data analysis revealed that there were significant differences between clinical and nursing specialties (3.35 ± 0.3) and others, but there was no significant difference between nursing and others. Another study found that rehabilitation nursing majors significantly performed lower than their counterparts in the nursing and midwifery majors (16).

PI fluctuates significantly in different grades of medical students, and clinical practice is a crucial factor to PI performance (17). Initially, when students enter medical school, their PI is at its peak, as they perceive their PI as “given to” them by society. However, from the second year, their PI performance declines. Increased professional knowledge and abilities do not improve the PI of medical students. After clinical practice for internship, PI starts to rebound visibly (16,18). However, after experiencing the severe pressure of COVID-19 during clinical practice, the PI increases only slightly, compared with their performance before the internship (15).

The development of the PI in medical students is a dynamic process influenced by multidimensional factors, which causes them to reconsider pursuing a career in medicine in accordance with their individual perceptions. The most frequently analyzed factors are the basic information regarding the students, including age, gender, academic grade, and residential area (15,18,19,20). Other influencing factors can be divided into four categories: personal, family, professional characteristics, and social factors (15,16,18-20).

Notably, COVID-19 has been a new factor impacting PI since its outbreak. On the one hand, to adjust to the changes brought about by the epidemic, rotations were canceled, and practice was occasionally turned into online theory learning for medical students in their practical year. On the other hand, medical interns were occasionally involved in heavy work related to controlling and preventing epidemics. In light of the temporary crisis caused by COVID-19 and the increasing number of graduates, there is a vacuum in the study of PI among new rehabilitation graduates, who completed nearly all of their university coursework and their internship during the outbreak.

More than 100,000 new graduates get a degree of rehabilitation therapy each year in China, and this number is steadily increasing (21). Nonetheless, the rehabilitation industry has experienced ‘a cold winter’ in China because of COVID-19. First, many essential rehabilitation therapy programs have been removed from medical insurance, in accordance with the most recent policy (22). Second, the rehabilitation business in China has subsequently witnessed a significant decline in employment and output (23). A few rehabilitation businesses had to suspend operations, while others were able to continue operations by reducing expenses, including the salaries of therapists (24). Last but not least, the overburdened healthcare system instilled a sense of fatigue in rehabilitation therapists due to COVID-19. Overall, the adverse professional environment may prompt rehabilitation graduates to reconsider their career options, which might have consequences for their professional identities.

In sum, the influence of COVID-19 on the healthcare system, the allocation of medical resources, and the integration of employees has weakened the rehabilitation business. From the standpoint of higher education, PI represents the level of recognition of rehabilitation graduates and the development of the rehabilitation business in the post-epidemic age. This study evaluated the professional identity of new graduates majoring in rehabilitation therapy during the final harsh phase of COVID-19 and explored the factors influencing PI.

## Materials and Methods

**Questionnaire design**: We used the Professional Identity Questionnaire for Students (PIQS) for our survey (Table 1), which was validated and reliable in previous research (25) utilized by numerous professional research (26,27). It consists of 23 self-administered questions that assess four dimensions of professional identity: cognition (five questions, numbered 1-5), emotionality (eight questions, numbered 6-13), behavior (six questions, numbered 14-19), and fitness (four questions, numbered 20-23). These dimensions are cognition, emotionality, behavior, and suitability. Cognition refers to the fundamental knowledge of the profession, emotionality to an emotional attachment for the profession, behavior to conduct within the profession, and suitability to the degree of compatibility between the profession and the personality traits of students. In addition to PI, their demographic and descriptive information was also collected. Age, gender, status of residence, employment, employment patterns, and educational patterns were among the demographic and descriptive characteristics provided.

**Table 1:** Professional identity questionnaire for students

S.N	Questions
1.	I understand the requirements of my major for students.
2.	I understand the employment conditions for graduates in my major.
3.	I know the ranking of my major at my university.
4.	I know what the outside world thinks of my major.
5.	I understand my major in general.
6.	I am willing to work in a job related to my major.
7.	I have embraced my major from the bottom of my heart.
8.	I have never thought about changing my major.
9.	I would give a positive evaluation of my major.
10.	I have full confidence in the future of my major.
11.	I have developed positive emotions towards my major.
12.	I am very satisfied with the overall situation of this university and this major.
13.	I like my major in general.
14.	I often read books related to my major.
15.	I complete assignments for courses in my major carefully and on time.
16.	I pay attention to the courses in my major.
17.	I spend a lot of time on my major.
18.	I am persistent in the study of my major.
19.	I actively participate in practical activities related to my major.
20.	I possess good professional thinking skills.
21.	My personality matches my major.
22.	My major reflects my expertise.
23.	I feel at ease with this major.

**Participants and data collection**: Convenience and nonprobability samplings were used in sample selection. The participants were recruited between May and June of 2022 in China. They were volunteers and were not given any monetary compensation. The inclusion conditions required students to be conscious, at least 18 years old, proficient in Chinese, rehabilitation therapy major graduates, and able to provide informed consent. In addition to pregnant students, the exclusion criteria were participants with a history of drug misuse, criminal behavior, or other specified situations. A self-administered questionnaire was distributed to liaison personnel at hospitals, universities, and other institutions by paper or electronic methods, to adapt to a variety of contexts.

The program was popularized by numerous rehabilitation treatment practitioners. Contacts were established in the rehabilitation departments of various hospitals and in the rehabilitation specialties of higher education. The contact person would send the questionnaire to the new graduates of the rehabilitation major for completion, and thoroughly explain the contents before the participants filled it out. They would also offer clarifications as needed to ensure accurate responses to the questions. To guarantee the accuracy of the data, information from each site was gathered from contacts there, compiled and tabulated, and then double-checked by two researchers.

**Score calculation and interpretation**: Each item was rated as follows on a 5-point Likert scale (1-strongly disagree, 2-disagree, 3-neutral, 4-agree, 5-strongly agree). We determined the mean response score for each question so that the study would be statistically sound and understandable, based on an original study (17). Therefore, the overall performance was the average score of all 23 items in the questionnaire. In addition, scores of cognitions (question 1-5), emotionality (questions 6-13), behavior (questions 14-19), and fitness (questions 20-23) were calculated to correspond to the four dimensions of content characteristics.

**Data analysis**: Statistical analysis was conducted using SPSS version 22.0 (IBM Corporation, Armonk, New York, USA) and Microsoft Office Excel version 2007 (Microsoft Corporation, Redmond, Washington, USA). Ranges, means, and standard deviations (SD) were calculated using descriptive statistics, whereas frequency and percentage were utilized for categorical data. Nonparametric test was used to assess the differences of PI based on the research variables of gender, residential status, employment, and educational patterns. Analysis of variance was used to compare differences of PI based on employment patterns. univariate linear regression and multiple linear regression analyses were used to filter out the potential predictors of professional identity among five variables—age, gender, status of residence, employment, employment patterns, and educational patterns. P < 0.05 was considered significant for all tests.

**Ethical approval**: Ethical approval was granted from the Committee on Biomedical Ethics of Guangdong Work Injury Rehabilitation Hospital (AF/SC-07/2022.17). This study ensured the anonymity and confidentiality of all collected data, and the voluntary involvement of all respondents.

## Results

Rehabilitation students from 99 higher education institutions (65 four-year universities, 34 three-year colleges) in 25 provinces or cities having provincial status responded to the questionnaires. However, there was little unevenly distributed data in each area. Finally, 786 respondents were included in the study, and 780 of them (with a response rate of 99.23%) completed the entire questionnaire.

**Figure 1:**
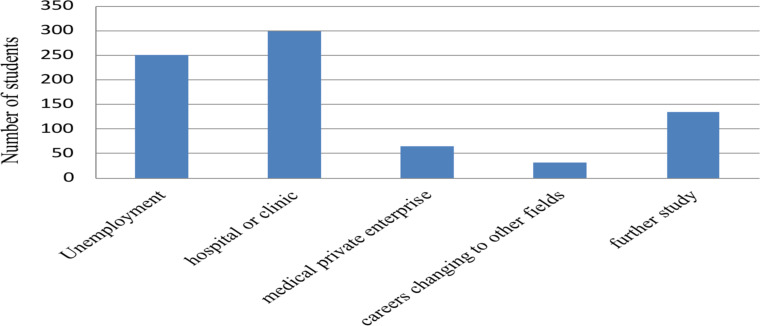
Classification of employment

**Demographics and descriptive information**: Of the 780 participants, 30.3% were males and 69.7% were females. The age range was 18 to 30, with a mean of 22.47 years (SD = 2.00). The majority of the participants (61.5%) came from rural villages or towns. Upon graduation, 67.8% of the participants had received offers for jobs or further studies. Of the employed participants, the majority obtained jobs in hospitals or clinics (38.3%), followed by those pursuing additional education (17.3%), and medical private enterprise (8.2%). A total of 4.0% of the students changed their academic major (Figure 1). In addition, the ratio of participants attending three-year colleges to those attending four-year universities was 1:2.04 (Table 2).

**Table 2:** Demographics and descriptive information

Descriptive	Characteristics		

	N (%)	Mean (SD)/Median (IQR)	P
Gender			
Male	236 (30.3%)	3.91 (3.61, 4.22)	0.15
Female	**544** (69.7%)	3.87 (3.57, 4.04)	
Age	/	22.47 (2.00)	/
Status of residence			
Rural	480 (61.5%)	3.87 (3.57, 4.08)	0.87
Urban	300 (38.5%)	3.85 (3.53, 4.13)	
Employment			
Unemployed	251 (32.2%)	3.83 (3.39, 4.00)	0.003
Employed	529 (67.8%)	3.87 (3.61, 4.17)	
Employed patterns			0.04
Unemployment	251(32.2%)	3.83 (3.39, 4.00)	
Hospitals or clinics	299(38.3%)	3.91 (3.61, 4.17)	
Medical private enterprise	64(8.2%)	3.87 (3.65, 4.17)	
Further study	31(4.0%)	3.91 (3.39, 4.26)	
Career change to other fields	135(17.3%)	3.87 (3.60. 4.08)	
Educational patterns			
three-year colleges	254 (32.6%)	3.91 (3.65, 4.30)	0.001
four-year universities	519 (66.5%)	3.83 (3.52, 4.00)	
Not answered	7 (0.9%)		
Total	780 (100%)		

N: number; SD: standard deviation; IQR: Interquartile range

**Professional identity**: Starting with the overall performance, the average PI score was 3.84, although the scores of the four dimensions ranged around this value. The average scores for cognition (3.95±0.54) and behavior (3.93±0.60) were close to 4, whereas fitness had the poorest performance (3.64±0.74). The emotionality performance (3.80±0.71) was closest to the mean.

There was no significant difference of PI between males and females, and the PI of status of residence between rural and urban. However, employed graduates showed better performance on the PI than unemployed ones. In addition, graduates of three-year institutions had higher PI scores than those of four-year universities. There was a significant difference of PI among different employed patterns, and the main patterns were for unemployment. The only significantly different group in terms of employment patterns was the unemployed, which performed the worst compared to other groups.

Univariate regression was computed to filter out the potential predictors of PI. Two factors, employment and educational patterns, were included. Multiple linear regression was then used to inspect these predictors and build the predicting model. Definitely, the PI was statistically predicted by two factors, employment (Beta= -0.09, P< 0.05) and educational patterns (Beta= 0.12, P< 0.01), with a R^2^ of 0.02 (F (2, 770) = 7.99, P< 0.01) (Table 3).

**Table 3:** Results of linear regression analysis

Variable	Univariate regression Beta (95% CI)	Multiple Linear regression Beta (95% CI)
Gender		
Male	0.03 (-0.06, 0.11)	
Female	Referance	
Employment		
Unemployment	-0.14(-0.22, -0.05)**	-0.09 (-0.18, -0.004)*
Employed	Referance	
Employed patterns		
Unemployment	-0.12 (-0.24, 0.001)	
Hospitals or clinics	0.02 (-0.09, 0.14)	
Medical private enterprise	0.04 (-0.13, 0.21)	
Further study	0.03 (-0.02, 0.25)	
Career change to other fields	Referance	
Status of residence		
Urban	-0.01(-0.09, 0.08)	
Rural	Referance	
Educational patterns		
Three-year colleges	0.15(0.06, 0.23)**	0.12 (-0.03, 0.21)**
Four-year universities	Referance	
Age	-0.006 (-0.03, -0.02)	

CI, confidential interval

* P<0.05

** P<0.01

## Discussion

Despite the fact that numerous studies explored PI development among medical students, few focused on new rehabilitation graduates and influencing factors to measure the PI levels post-COVID-19. This study investigated the professional identity of rehabilitation students in relation to the impact of COVID-19 during the final phase of prevention and control, and to explore influencing factors associated with PI. Two main findings were obtained (1). The average PI was still quite a distance from the degree of ‘agree,’ particularly for the fitness dimension, and (2) performance of PI was associated with employment and educational patterns. The findings not only expand the knowledge and understanding of professional identity but can also serve as a guide for the education and management of the health system during other potential outbreaks in the future. For instance, it can assist managers to improve organizational justice and adjust policies on the distribution of medical resources in response to COVID-19-induced structural changes in the rehabilitation industry.

The average PI was still quite a distance from the degree of ‘agree.’ On a 5-point Likert scale, the average PI score was 3.84, which was lower than 4, the ‘agree’ grade, although it was much higher than the performance of medical students in a 2009 study (about 3.1) (17) and another study that focused on the medical students post-COVID-19 in 2022 (about 3.4) (15). On the positive side, a possible explanation for the improvement of PI may be that rehabilitation has been gradually accepted by students as a consequence of the ten-year expansion of the rehabilitation industry. Since the Wenchuan earthquake of 2008, the rehabilitation industry has witnessed a substantial shortage of specialists. Nearly each municipality and street has developed federations of disabled citizens (28,29). The development of rehabilitation is still in its infancy and shows great promise for anyone interested in pursuing it as a career.

On the negative side, however, the performance of PI still requires to enhancement. Consequent to the rapid development of the rehabilitation industry, rehabilitation practitioners have to undertake high-intensity work due to the consequences of population aging and changes in the disease spectrum, particularly the current threat of COVID-19. This situation may have impacts on their professional beliefs, attitudes, and personal values, and thereby influencing potential labor in the rehabilitation industry (5).

Another point of particular concern was the fitness dimension, which had the lowest performance (3.64) compared to others. Fitness manifests if the students believe they are qualified for employment in the rehabilitation business. Medical students who have completed their internships are more likely to regret their career choice compared to those who have not yet begun their clinical internships (30). It may be because professionals, including rehabilitation interns, actively took part in the treatment or nursing of COVID-19 patients during the outbreak. They might even have been required to assist neighborhood-based epidemic prevention efforts. In addition, another harsh truth was that the COVID-19 outbreak had occupied them for the entirety of their internship. Consequently, it was likely that these rehabilitation interns were under great emotional and physical strains, and witnessed the overloading of medical staff, which made them reconsider their career choice and for employment in the rehabilitation business.

Regarding the influencing factor of employment, PI tends to fade in an unemployed state and terrible work context (31). In line with the findings of previous research, the results of this study revealed that the performance of professional identity was significantly associated with employment. Employed individuals performed better in terms of PI than unemployed participants. A possible explanation may be that these unemployed students were pushed to reconcile with aspects of their PI and the decline of the rehabilitation job market, and then had to reassess their professional career choice and PI. This survey showed that the employment rate was disturbingly low at 67.8%. The escalation of contradictions by COVID-19 compels the higher education sector to concentrate on both enduring issues and potential crises. On the one hand, there is no doubt that the reckless growth of rehabilitation in higher education, coupled with frenzied socioeconomic reforms and inadequate labor market research (32), exacerbated the crisis of unemployment and underemployment prior to 2020. On the other hand, following the pandemic, a considerable number of well-educated rehabilitation graduates returned (33), while the rehabilitation industry contracted (34-36), thus resulting in heightened competition in the domestic labor market. Eventually, tens of thousands of rehabilitation graduates were presented with a more competitive work climate with lower pay and increased performance standards (33). These findings supported the fact that higher education of rehabilitation should respond to the present shift in the structure of the rehabilitation industry in the wake of COVID-19.

In addition, this study demonstrated that performance of PI was significantly associated with educational patterns. Individuals who graduated from three-year colleges had higher scores of PI than those who graduated from four-year universities. It may stem from multiple reasons, including the trending policies of the rehabilitation industry and tendencies in China, and individuals' efficiency of educational investment. In China, two types of institutions offer higher education: research universities and practice-oriented colleges. The level of education, entry criteria, and final graduate certifications are generally lower in colleges compared with those in universities. It takes three years for college graduates to achieve a bachelor's degree, and four years for university graduates. However, for both types of rehabilitation graduates, the professional certification necessary, available positions, and salaries are almost the same. This discrepancy may lead to a higher expectation of university graduates and a different performance in PI due to higher requirements for admission and more years to complete the course. For another, colleges appear easier to access for small and medium-sized enterprises, because of their focus on business practices (37). Numerous policies have been implemented to facilitate the transfer of theoretical knowledge and production practice in vocational education sectors in China, such as “Integration of industry and education” production and “Combination of work and study” internships (38). This vocational training in colleges has made rehabilitation graduates more adaptable to a wide range of small and medium-sized enterprises and grass-roots posts.

In conclusion, the overall performance of PI of graduates still needs to be promoted, after facing the challenge of COVID-19, particularly the dimension of fitness. Higher education on rehabilitation should respond to the present shift in the structure of the rehabilitation industry, following COVID-19. Meanwhile, the occupational environment of rehabilitation therapists and the medical value of rehabilitation therapy deserve a great deal of attention. However, this study had some limitations. First, this cross-sectional study was without follow-up after graduating students to identify any alterations. Second, a self-evaluation questionnaire was used, so the answers may not fully reflect the true thoughts of students. In the future, we will regularly monitor the PI changes of post-graduation rehabilitation graduates and conduct in-depth interviews to uncover hidden details.
